# A 3D cephalometric protocol for the accurate quantification of the craniofacial symmetry and facial growth

**DOI:** 10.1186/s13036-019-0171-6

**Published:** 2019-05-17

**Authors:** Manuel Pinheiro, Xinhui Ma, Michael J. Fagan, Grant T. McIntyre, Ping Lin, Gautham Sivamurthy, Peter A. Mossey

**Affiliations:** 1 School of Engineering and Computer Science, University of Hull, Hull, UK; 2 Department of Orthodontics, School of Dentistry, University of Dundee, Dundee, UK

**Keywords:** Craniofacial morphology, Facial symmetry, Facial growth, Cephalometry, Phantom study

## Abstract

**Background:**

Cephalometric analysis is used to evaluate facial growth, to study the anatomical relationships within the face. Cephalometric assessment is based on 2D radiographic images, either the sagittal or coronal planes and is an inherently inaccurate methodology. The wide availability of 3D imaging techniques, such as computed tomography and magnetic resonance imaging make routine 3D analysis of facial morphology feasible. 3D cephalometry may not only provide a more accurate quantification of the craniofacial morphology and longitudinal growth, but also the differentiation of subtle changes in occlusion. However, a reliable protocol for the computation of craniofacial symmetry and quantification of craniofacial morphology is still a topic of extensive research. Here, a protocol for 3D cephalometric analysis for both the identification of the natural head position (NHP) and the accurate quantification of facial growth and facial asymmetry is proposed and evaluated. A phantom study was conducted to assess the performance of the protocol and to quantify the ability to repeatedly and reliably align skulls with the NHP and quantify the degree of accuracy with which facial growth and facial asymmetry can be measured.

**Results:**

The results obtained show that the protocol allows consistent alignment with the NHP, with an overall average error (and standard deviation) of just 0.17 (9.10e-6) mm, with variations of 0.21 (2.77e-17) mm in the frontonasal suture and 0.30 (5.55e-17) mm in the most prominent point in the chin. The average errors associated with simulated facial growth ranged from 1.83 to 3.75% for 2 years’ growth and from − 9.57 to 14.69% for 4 years, while the error in the quantification of facial asymmetry ranged from − 11.38 to 9.31%.

**Conclusions:**

The protocol for 3D skull alignment produces accurate and landmark free estimation of the true symmetry of the head. It allows a reliable alignment of the skull in the NHP independently of user-defined landmarks, as well as an accurate quantification of facial growth and asymmetry.

## Introduction

Cephalometric analysis is used to evaluate facial growth, to study the anatomical relationships within the face and as a routinely used tool for treatment planning in orthodontics and craniomaxillofacial deformity surgery. A standard cephalometric assessment is based on 2D radiographic images taken in either the sagittal (lateral cephalogram) or coronal planes (posteroanterior cephalogram), where multiple landmarks, lines and angles are identified to quantify vertical and horizontal relationships in the face. The analysis is primarily dependent on the accurate and repeatable definition of a standardized head position, therefore patients are imaged in customized image acquisition systems [1]. Regardless of the screening protocol, cephalometric images may be affected by magnification artefacts, craniofacial asymmetry and the superimposition of anatomical structures, all contributing to the imprecise and inaccurate traditional evaluation methods of facial morphology.

2D cephalometrics is an inherently inaccurate methodology, regarding the quantification of small increments of facial growth and the analysis of the transverse dimensions, in particular the analysis of facial asymmetry. The accuracy of contemporary cephalometric analysis is further complicated by the variation, inconsistency and errors in image capture, image quality and landmark identification, as well as limited intra and inter observer reliability and reproducibility [2]. The inability to detect slight changes in craniofacial morphology limits the clinical application of this technique and hinders its research usefulness in areas such as the quantification of longitudinal growth or the differentiation of subtle changes in occlusion, such as intercuspal and retruded contact position. No technique to date offers a precise virtual reproduction of dental occlusion [3], with study models and facebow recordings being the current gold standard in dentistry for the reproduction of inter-occlusal relationships.

The wide availability of 3D imaging techniques, such as computed tomography (CT), cone-beam CT (CBCT) and magnetic resonance imaging (MRI), makes routine 3D analysis of facial morphology feasible. 3D reconstructed cephalometry possesses several advantages when compared with traditional cephalometry. It avoids the need for standardized fixation of the head and allows an a posteriori definition of the anatomical head position. It also prevents structure magnification and distortion inherent to imaging process, allowing at the same time the full assessment of the 3D craniofacial morphology, which is important for pre-operative evaluation, surgical planning and post-operative evaluation [1]. 3D imaging techniques have the potential to provide significant precision improvements, however the accurate translation of well-established 2D concepts to a 3D framework needs further development. Studies in 3D cephalometry show conflicting results, with some studies concluding that it provides a more accurate evaluation of the craniofacial anatomy [4–6], while others conclude that there is no statistical difference between the two techniques [7, 8], or that it is less accurate than its 2D counterpart [9].

The most critical aspect of 3D cephalometric assessment is the accurate positioning of the skull in space, requiring accurate definition of both the craniofacial symmetry plane and the appropriate anatomical frame for the head. In 2D analysis of the lateral cephalogram, the skull is positioned through the identification of the true horizontal or the true vertical direction, and the identification of the S, as the reference point for image superimposition and comparison. The Frankfurt horizontal (FH) plane is used to orient the lateral cephalometric image in some specific analyses, requiring the identification of two bilateral landmarks (porion, Po, and orbitale, Or), which do not lie in the same plane as assessed using 3D imaging due to the natural asymmetry of the human face. In addition, FH related landmarks are amongst the most subjective landmarks and therefore can be prone to erroneous identification [5, 10]. The alternative is to define the true vertical direction, such as the Nasion-Pogonion line [11]. This approach has the advantage of being based in the identification of the nasion (N) and pogonion (Pog), which are also less subjective than Po and Or [5], and may be regarded as lying in the symmetry plane of the head. These landmarks provide direct information about the midline structures of the face and are not subject to anatomical variation due to facial asymmetry. In addition, during childhood and adolescence, craniofacial growth is characterized by a relatively stable cranial base and foramen magnum, which remain relatively unchanged, when compared with the significant expansion of the cranial vault and distal and forward growth of both maxilla and mandible [1, 12, 13]. In a 3D setting, the basicranium can provide a reliable reference structure for further craniofacial growth assessment [1].

The development of a consistent and reliable 3D cephalometric workflow will provide clinicians with a valuable tool for the 3D characterization and quantification of facial growth, for the evaluation of dentofacial relationships and subsequent anatomically accurate orthodontic and surgical planning, and for the evaluation of treatment outcomes. We present a protocol for the accurate estimation of craniofacial symmetry, evaluating this using random skull orientations, facial growth vectors and different types of facial asymmetry. The null hypothesis tested was that the 3D method for analysing facial growth and asymmetry was not reliably quantifiable. The alternative hypothesis was that both craniofacial symmetry plane, facial growth and facial asymmetry could be accurately quantified with the proposed 3D algorithm.

## Materials and methods

A skull model was obtained from the GrabCAD database (https://grabcad.com/), and skull landmarking (Table 1) conducted in Avizo v9.4.0 (Visualization Science Group). The 3D landmark coordinates were exported for further analysis and morphing with MATLAB R2007b and RStudio, respectively.

**Table 1:** Standard cephalometric landmarks used for the geometric morphometric analysis of the human craniofacial anatomy [11]

Landmark	Description
Alare (Al)	The most lateral points on the nasal aperture in a transverse plane.
Anterior nasal spine (ANS)	The most anterior point at the sagittal plane on the bony hard palate.
Basion (Ba)	The most inferior posterior point of the occipital bone at the anterior margin of the occipital foramen.
Crista galli (Cg)	Most superior point on the crista galli.
Ectoconchion (Ec)	The intersection of the most anterior surface of the lateral border of the orbit and a line bisecting the orbit along its long axis.
Ectomolare (Ecm)	The most lateral point on the outer surface of the alveolar borders of the maxilla, often opposite the middle of the second molar tooth.
Gnathion (Gn)	The midpoint between the most anterior and inferior points of the hard tissue chin in the midsagittal plane.
Gonion (Go)	The most outward inferior point on the angle of the mandible.
Inferior nasal aperture (IN)	Most inferior point on the inner cortex of the anterior nasal aperture.
Menton (Me)	The most inferior midline point on the mandible.
Nasion (N)	The point of intersection between the frontonasal suture and the midsagittal plane.
Orbitale (Or)	The lowermost point in the lower margin of the bony orbit.
Porion (Po)	The most superior point of the external auditory meatus.
Pogonion (Pog)	The most prominent point in the chin.
Sella (S)	The geometrical centre of the sella turcica.
Subspinale (A)	The most concave point of anterior maxilla.
Supraorbital notch (So)	Most superior point on the inner cortical plate of the orbital rim.
Anatomical plane	Description
Frankfurt horizontal	A line connecting the Po and Or points.
Nasion-Pogonion	A line connecting the N and Pog points
Sella-Nasion	A line connecting the Sella and the Nasion points.

**Fig. 1:**
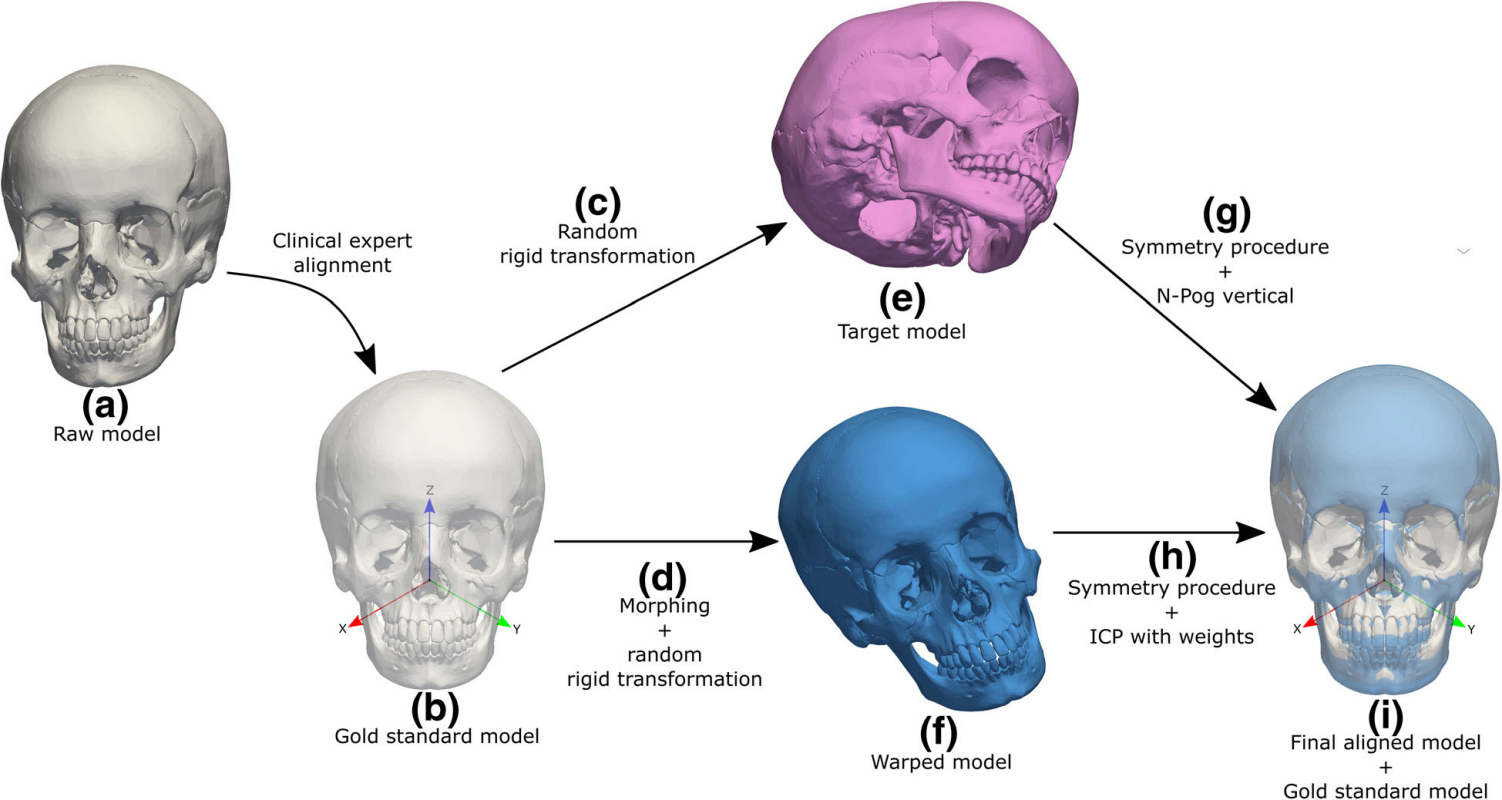
Diagram showing the main steps considered during testing of the new cephalometric protocol, in which (**a**) a *raw model* is aligned according to the natural head position (NHP) by a clinical expert to define (**b**) the *gold standard model*; next (**c**) a randomly generated rigid transformation or (**d**) mesh warping together with a random rigid transformation is applied to the *gold standard model* to produce several (**e**) target and (**f**) warped models, respectively; finally in (**g**) and (**h**) the craniofacial symmetry procedures are used to recover the ideal anatomical alignment (**i**)

To simulate different types of facial growth and asymmetry, different geometrical transformations were applied to the reference skull. Several definitions are used to distinguish the different models, namely:

- *raw model*: the initial/raw skull geometry obtained from the medical image data (Fig. 1a);
- *gold standard model*: the raw model aligned according to the natural head position (NHP) and landmarked by a clinical expert (Fig. 1b). The NHP is defined as the position that the head adopts when the patient is sitting or standing, looking to the horizon or at a distant object [14];
- *target model*: the gold standard model after random rigid transformation (Fig. 1e);
- *warped model:* the gold standard model after mesh warping and random rigid transformation (Fig. 1f).

Figure 1 provides a graphical representation of the workflow from the *raw model,* to the definition of the *gold standard model*, *target models* and the final alignment. The use of the NHP aims to provide a common ground between standard 2D cephalometric evaluation and the protocol proposed in this work. It is important to note that all *target models* and *warped models* are generated from the *gold standard model*, since this contains the correct anatomical orientation provided by the clinical expert.

### Computation of the symmetry plane

To determine the correct symmetry plane of the skull Principal Component Analysis (PCA) [15] and Iterative Closest Point (ICP) methods [16, 17] are used interchangeably. The craniofacial symmetry procedure is as follows:

1. Reposition the original model in space according to the mesh centre of mass;
2. Perform PCA over the model and reorient it according to the calculated eigenvectors ***λ***_*i*_ ∈ *R*^3^;
3. Compute the reflection that minimizes the root-mean-squared error (RMSE) of the Euclidean distances between the nearest points in the original and reflected models, along the *eigen-planes* defined in step 2;
4. Apply ICP to register the original and reflected mesh created in step 3;
5. Perform the PCA over the combined models from step 4 and reorient the original model according to the newly obtained eigenvectors ${\boldsymbol{\lambda}}_i^{new}\in {R}^3$.

In steps 1 and 2 a local reference frame that is independent of the acquisition frame is computed. Step 3 finds the first approximation to the symmetry plane; however, it is affected by the natural asymmetry of the skull. In step 4 the original and mirrored models are matched to create a new perfectly symmetrical model, which is used in step 5 to compute a new estimate of the symmetry plane. The original model is then oriented according to the ${\boldsymbol{\lambda}}_i^{new}\in {R}^3$, which is independent of any existing asymmetry in the skull model. It is important to note that step 4 is key to the accuracy of the procedure and is dependent on the alignment strategy [18]. A preliminary sensitivity test of the ICP method was therefore conducted. These results showed that *point-to-plane* alignment provides very accurate alignment between equal meshes (average error over 15 trials was just 8.81e-9 (9.09e-15) mm). ICP registration with *point-to-plane* alignment was therefore set as the default in step 4 (for more details the interested reader is referred to [16, 17]).

### Positioning of the skull in the NHP

Evaluation of craniofacial morphology and growth is quantified considering a standardized anatomical position and therefore recovery of the NHP of the raw model may provide a consistent framework for 3D cephalometric assessment in clinical practice. The NHP can be defined by two landmarks along the symmetry plane, for example N and Pog, with the subsequent definition of the N-Pog line as the true vertical direction. However, it is important to note that use of the N-Pog line is not mandatory and two other landmarks along the symmetry plane could also be used to define the true vertical direction (for instance, N and A if the mandible is not available).

To assess the method’s reliability in recovering the NHP, a sensitivity test was performed. Rigid transformations were randomly generated and applied to the *gold standard model* (Fig. 1c). The rigid transformations consisted of random displacements −50 ≤ 〈*d*_*x*_, *d*_*y*_, *d*_*z*_〉 ≤ 50 mm and rotations along the three orthogonal directions −50 °  ≤ 〈*θ*_*x*_, *θ*_*y*_, *θ*_*z*_〉 ≤ 50°. The *target model* was then re-aligned with the *gold standard model* (Fig. 1g and i). This process was repeated 15 times and tested considering full (with cranial vault) and partial (no cranial vault) skull data, as in a standard CBCT acquisition process.

### Craniofacial growth and asymmetry

**Fig. 2:**
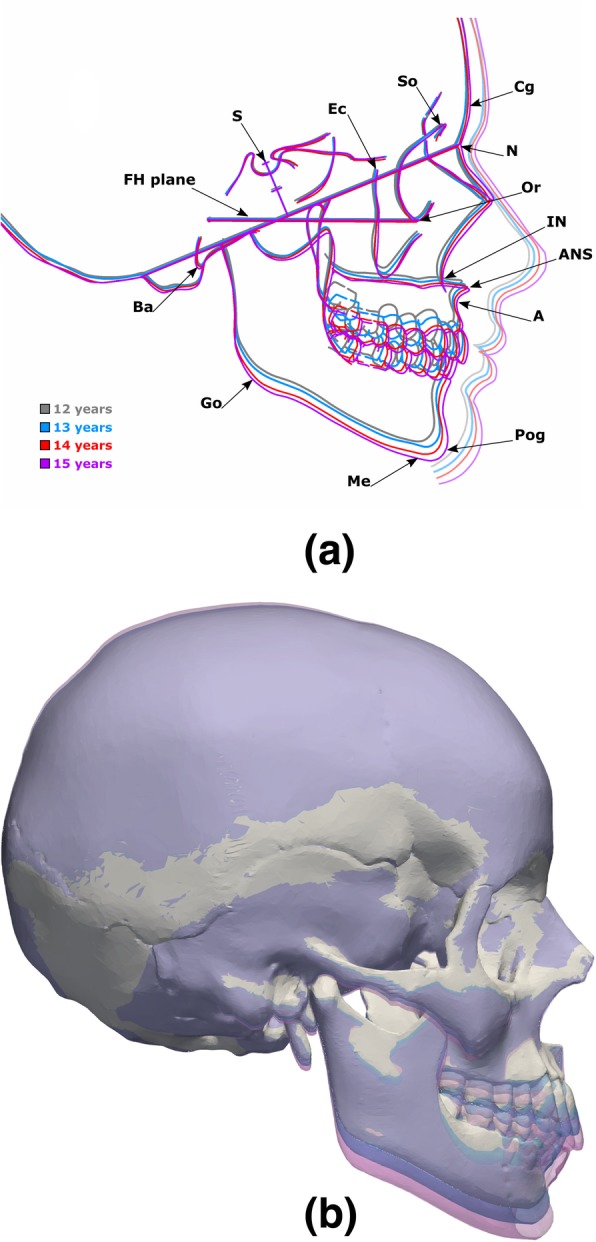
(**a**) average craniofacial growth between 12 to 15 years (adapted from [13]) with the main cephalometric landmarks used in craniofacial morphology assessment, and (**b**) ideal gold standard and target model alignment with theoretical 2-year (blue) and 4-year (purple) facial growth geometries

**Fig. 3:**
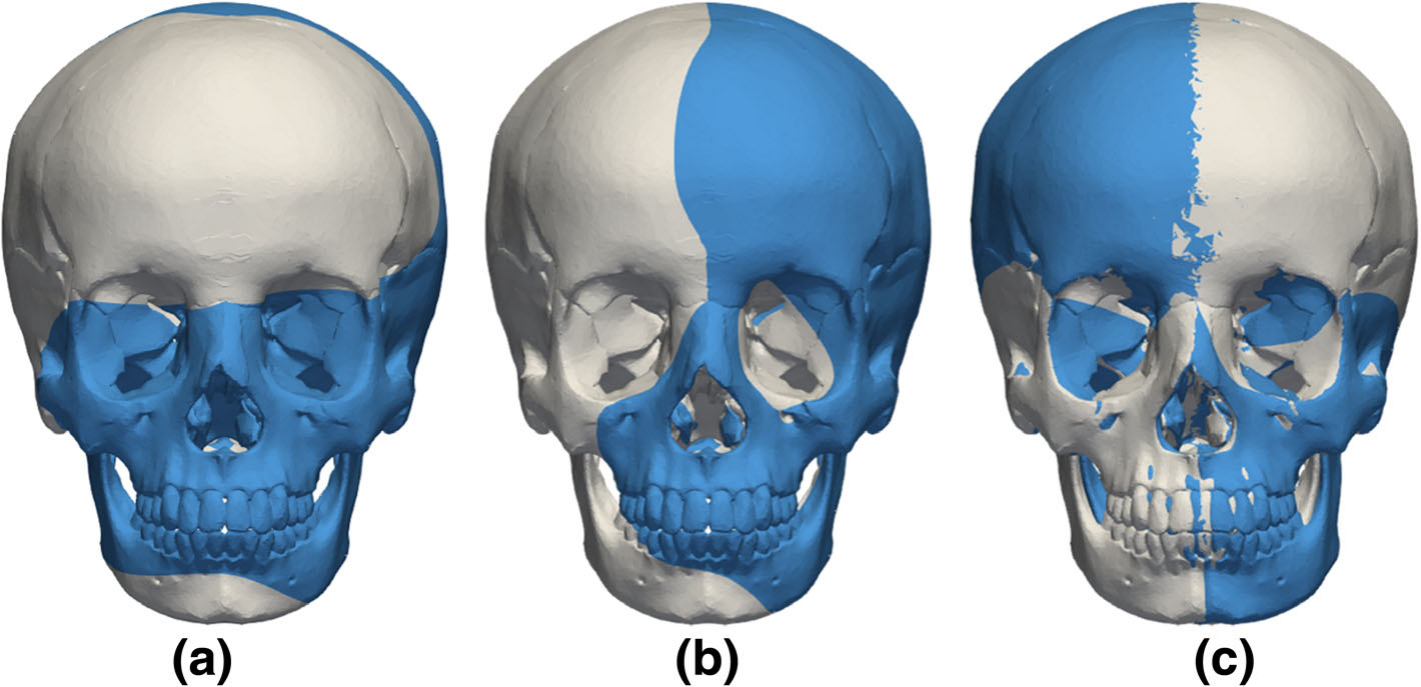
Modelling of facial asymmetry with: **a** protrusion of the midface, **b** unilateral protrusion of the face and (**c**) lateral displacement of the mandible (gold standard model in grey and asymmetric models in blue)

Craniofacial growth was simulated by warping the viscerocranium of the gold standard craniofacial model while keeping the neurocranium unchanged. Figure 2a shows the average craniofacial growth of male and female subjects between 12 to 15 years old (adapted from [13]). In normal craniofacial development, an anterior growth of approximately 5.0 mm and distal growth of typically 20.0 mm was observed in subjects aged between 10 to 18 years of age [19]. For the purpose of this study average anterior and distal growth rates of 0.625 mm/year and 2.50 mm/year were considered, respectively. The positions of Cg, N, So, Or, Ec and Ba were fixed, while Go was displaced downwards and the A, Pog and Me landmarks were displaced both forward and downward to match the desired age (Fig. 2b). Similarly, facial asymmetry was included by applying different displacements to the craniofacial landmarks, such as N, IN, A, Ec, Go and Or. Three types of facial asymmetry were considered: (1) protrusion of the midface where IN, A, left and right Or were displaced forward (Fig. 3a); (2) unilateral protrusion of the face where IN, A, left Ec were displaced forward and the right Or was displaced backward (Fig. 3b); and (3) lateral displacement of the mandible where A, Pog, left and right Go were displaced laterally (Fig. 3c).

Mesh warping for both facial growth and facial asymmetry transformations was applied to the gold standard model and computed using two geometric morphometric packages, namely *Geomorph* [20] and *Morpho* [21]. Since the basilar surface remains relatively unchanged from adolescence to adulthood [12] and is independent of the landmarks used in cephalometric assessment, it was used as the reference anatomical structure to guide craniofacial alignment of all skulls.

Quantification of growth patterns and facial asymmetries is clearly dependent on accurate superposition of the craniofacial scans corresponding to the different stages of development. Alignment of morphologically different scans can be accomplished through the following steps:

1. Computation of the symmetry plane as described in section 2.1;
2. Definition of the NHP as described in section 2.2;
3. Alignment refinement using IPC with weights (***w***_*i*_ ∈ *R*) assigned to the basilar surface (Fig. 1h and i).

By using ICP with weights, the error along the basilar surface is minimised. In point-to-plane alignment with weights, the weights are set to ***w***_*i*_ ∈ {0, 1}, thereby acting as a binary variable that only allows alignment of the points belonging to the basilar surface region. It is important to note that only translations along the symmetry plane are considered, in order to preserve any existing asymmetries computed with the procedure proposed in section 2.1.

### Evaluation measures

Evaluation of the cephalometric alignment for NHP and craniofacial growth and asymmetry was performed considering several measures, namely the alignment and distances between cephalometric landmarks against their respective landmarks on the *gold standard* model and general alignment measures such as the Hausdorff distance (*Hd*), where:

1 $$ Hd\left(\boldsymbol{A},\boldsymbol{B}\right)=\mathit{\max}\left[\underset{A}{\min}\left(\boldsymbol{d}\left(\boldsymbol{A},\boldsymbol{B}\right)\right),\underset{B}{\min}\left(\boldsymbol{d}\left(\boldsymbol{B},\boldsymbol{A}\right)\right)\right] $$

and the mean symmetrical distance (*MSD*)

2 $$ MSD\left(\boldsymbol{A},\boldsymbol{B}\right)=\frac{1}{m+n}\left[{\sum}_{i=1}^m\mathit{\min}\left(\boldsymbol{d}\left({\boldsymbol{a}}_{\boldsymbol{i}},\boldsymbol{B}\right)\right)+{\sum}_{j=1}^n\mathit{\min}\left(\boldsymbol{d}\left({\boldsymbol{b}}_{\boldsymbol{j}},\boldsymbol{A}\right)\right)\right] $$

where ***d*** stands for the 3D Euclidean norm and *m* and *n* are the number of points in ***A*** and ***B***, respectively [22].

In facial growth and asymmetry tests, there is no longer an exact correspondence between cephalometric landmarks. Since mesh warping was applied to the *gold standard model*, the ideal alignment between gold standard and warped mesh was considered to be the initial alignment after warping and before any rigid transformation (Fig. 1d). In both cases, the goodness of alignment between the *gold standard* and the *warped models* was quantified by comparing the realigned models with their corresponding non-transformed gold standard models (initial alignment after warping). In the evaluation of alignment of morphologically different skulls (section 2.3), the *Hd*, *MSD* and the deviation between the N and Pog were computed. To understand how much information about facial growth and asymmetry is preserved after transformation and realignment, the distances between *gold standard* landmarks and morphed landmarks were therefore also calculated.

## Results

**Fig. 4:**
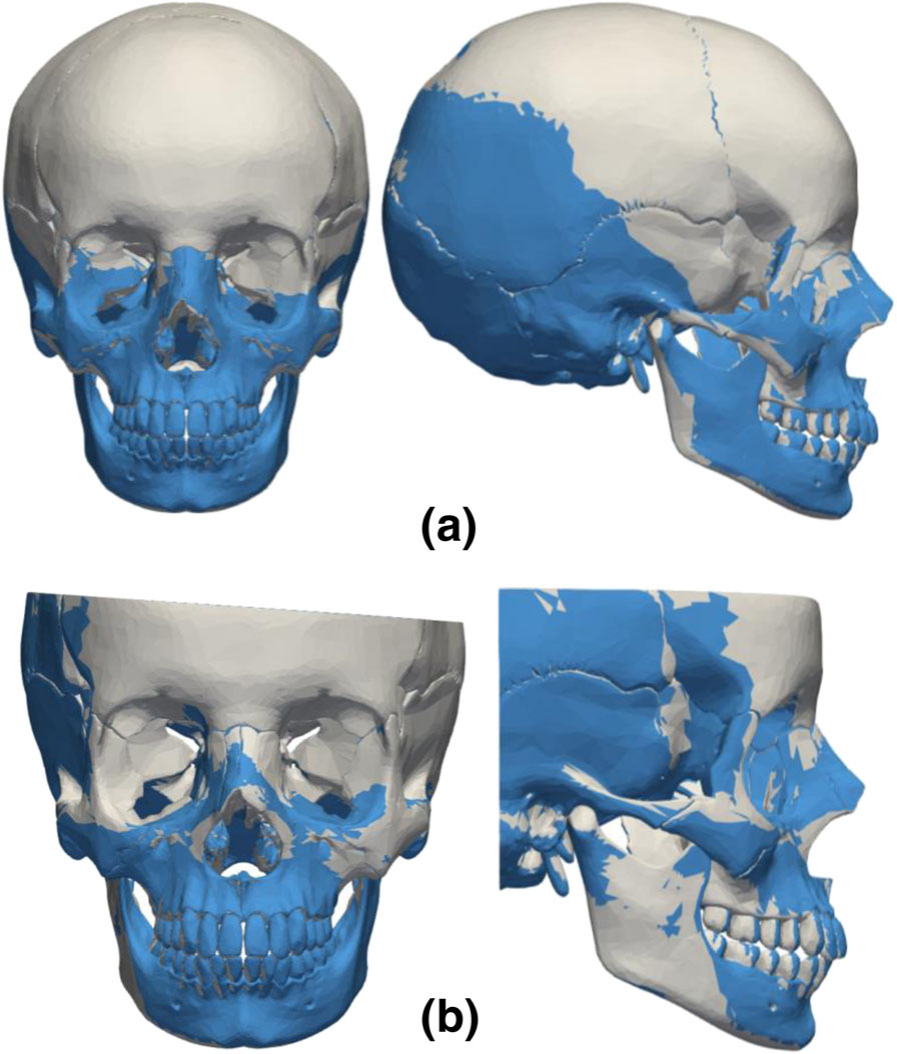
Recovering of the symmetry plane after: **a** coronal and sagittal alignment between the gold standard (grey) and target (blue) models in the presence of the full skull data, and (**b**) coronal and sagittal alignment of both gold standard (grey) and target (blue) in the presence of partial skull data

The reliability in the recovery of the NHP was tested by applying a random rigid transformation (Fig. 1c) to the target model (Fig. 1g) and comparing its calculated NHP with the gold standard model (Fig. 1i). The process was repeated 15 times and the results are summarized in Table 2. The *MSD* and *Hd* (with standard deviations) between the gold standard and target models are 0.17 (9.10e-6) mm and 0.52 (4.65e-5) mm for the full data, respectively. The true vertical was recovered with an average error of just 0.21 mm for the N and 0.30 mm for the Pog landmarks. For partial data, the *MSD* and *Hd* were 0.27 (2.23e-5) mm and 0.52 (4.26e-5), whereas the N and Pog landmark error were 0.32 (2.63e-7) and 0.23 (1.76e-7), respectively. Figure 4 shows the original transformation and subsequent realignment between the gold standard model and the target model after estimation of the NHP for the full skull (Fig. 4a) and partial skull data (Fig. 4b).

**Table 2:** Alignment errors obtained in the alignment of the target models (NHP recovery) and warped models (facial growth and asymmetry) with the *gold standard* models (*Mean Symmetrical Distance* (MSD), *Hausdorff distance* (Hd), and landmark errors in millimetres)

		N. trials	Aligned	MSD (sd)	Hd (sd)	Landmarks (sd)
NHP recovery	Full	15	15	0.167 (9.10e-6)	0.525 (4.65e-5)	*N* = 0.211 (2.77e-17) Pog = 0.305 (5.55e-17)
	Partial	15	15	0.272 (2.23e-5)	0.521 (4.26e-5)	*N* = 0.320 (2.635e-7) Pog = 0.232 (1.764e-7)
Facial growth		2	2	0.182 (0.062)^#^	0.337^#^	*N* = 0.091^#^ Pog = 0.162^#^
				0.696 (0.331)^*^	1.813^*^	*N* = 1.673^*^ Pog = 1.790^*^
Asymmetry		3	3	0.093 (0.035)	0.173	*N* = 0.106^**^ Pog = 0.173^**^
				0.201 (0.074)	0.372	*N* = 0.248^***^ Pog = 0.370^***^
				0.242 (0.136)	0.572	*N* = 0.022^##^ Pog = 0.079^##^

^#^Distance between the target model and the theoretical gold standard for 2-year growth; ^*^Distance between the target model and the theoretical gold standard for 4-years growth; ^**^Distance in facial asymmetry Fig. 3a; ^***^Distance in facial asymmetry Fig. 3b; ^##^Distance in facial asymmetry Fig. 3c; sd = standard deviation

**Fig. 5:**
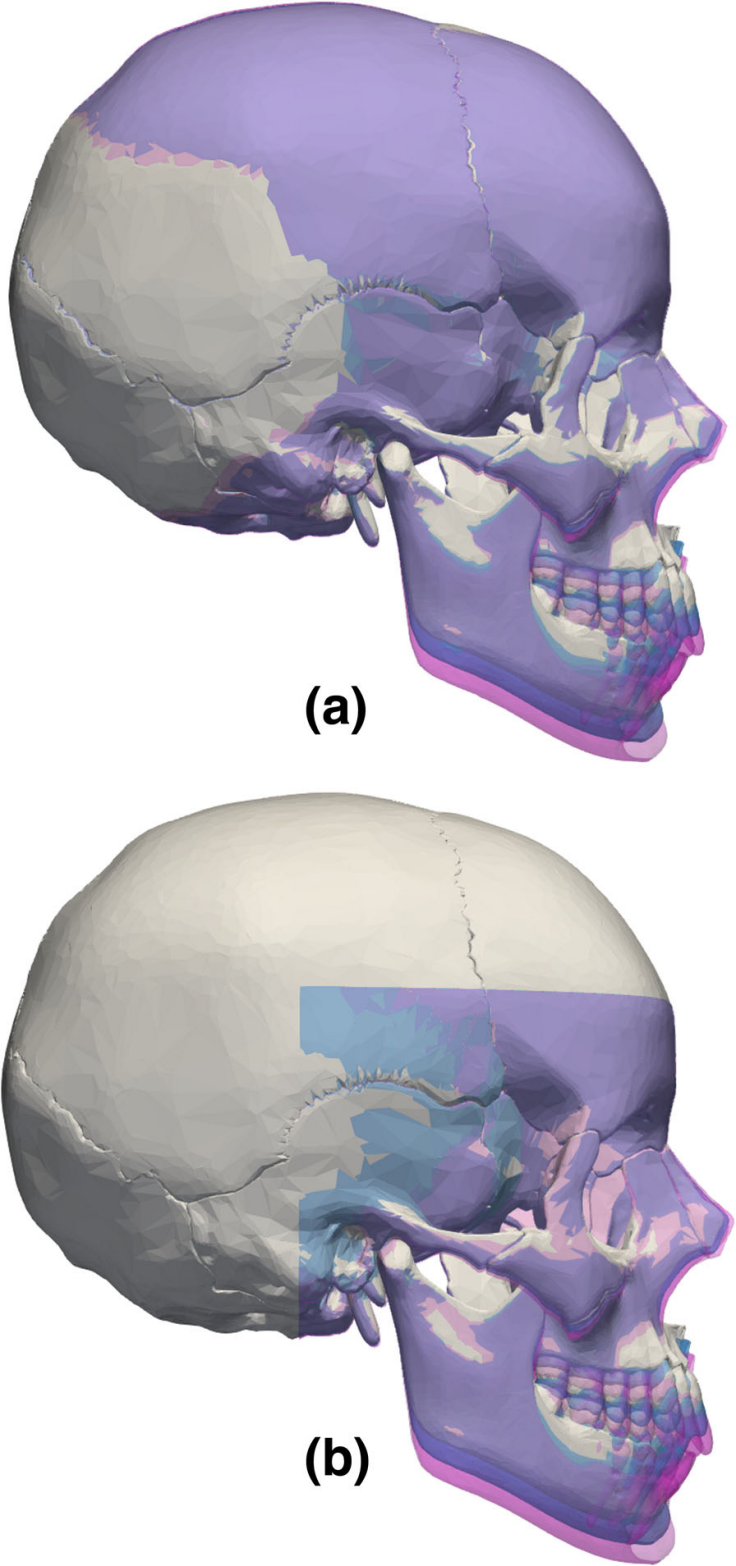
Final craniofacial time-series alignment: **a** after random transformation and realignment with full skull data, and (**b**) final alignment of the craniofacial time-series with partial skull data

The ability to quantify facial growth and asymmetry were tested by considering theoretical growths of 2 and 4-years (Fig. 2a) and three different types of facial asymmetry (Fig. 3a-c). The algorithm was able to re-align the warped models with their corresponding gold standard geometries (Fig. 5a-b). The N-Pog line was also recovered accurately, with an error of only 0.09 mm in the N and 0.16 mm in the Pog landmarks for the 2-year growth. For the 4 years data the error was 1.67 mm for the N and 1.79 mm for the Pog. The algorithm also performs well in the presence of partial skull data (Fig. 5b).

Table 3 shows the differences between the morphed landmarks from the gold standard and the 2-year and 4-year data before and after transformation. There is an overall overestimation of the distances between the gold standard and target model(s). For instance, before rigid transformation the distance A-A* between point A in the gold standard and the point A in the 2-year (warped gold standard) model is 4.97 mm. After rigid transformation and re-alignment, the distance A-A* is 5.06 mm, which corresponds to an overestimation of 0.091 mm. In the simulated 4-year facial growth, the alignment error increases and the distance A-A* is again overestimated by 1.61 mm (Table 3). The error between the theoretical and the quantified facial growth ranges between < 5% error for 2-years to < 15% for 4-years facial growth (Table 3).

**Table 3:** Distance between displaced landmarks in the gold standard model and the facial growth warped models (all values in millimetres)

	Distance	rGo-rGo^a^	lGo-lGo^a^	A-A^a^	Pog-Pog^a^	Me-Me^a^
2 years	Initial	4.9994	4.9995	4.9686	5.1538	5.1596
	Final	5.1869	5.1482	5.0596	5.2642	5.2789
	Diff.	0.1875	0.1487	0.0910	0.1103	0.1193
	%	3.75	2.97	1.83	2.14	2.31
4 years	Initial	10.6479	9.9990	10.9329	10.4213	10.3195
	Final	9.6290	10.9957	12.5389	11.9160	11.7514
	Diff.	−1.0189	0.9967	1.6060	1.4947	1.4319
	%	−9.57	9.97	14.69	14.34	13.87

^a^Landmarks in the theoretical 2-year and 4-year growth models; rGo stands for right gonion and lGo for left gonion

**Fig. 6:**
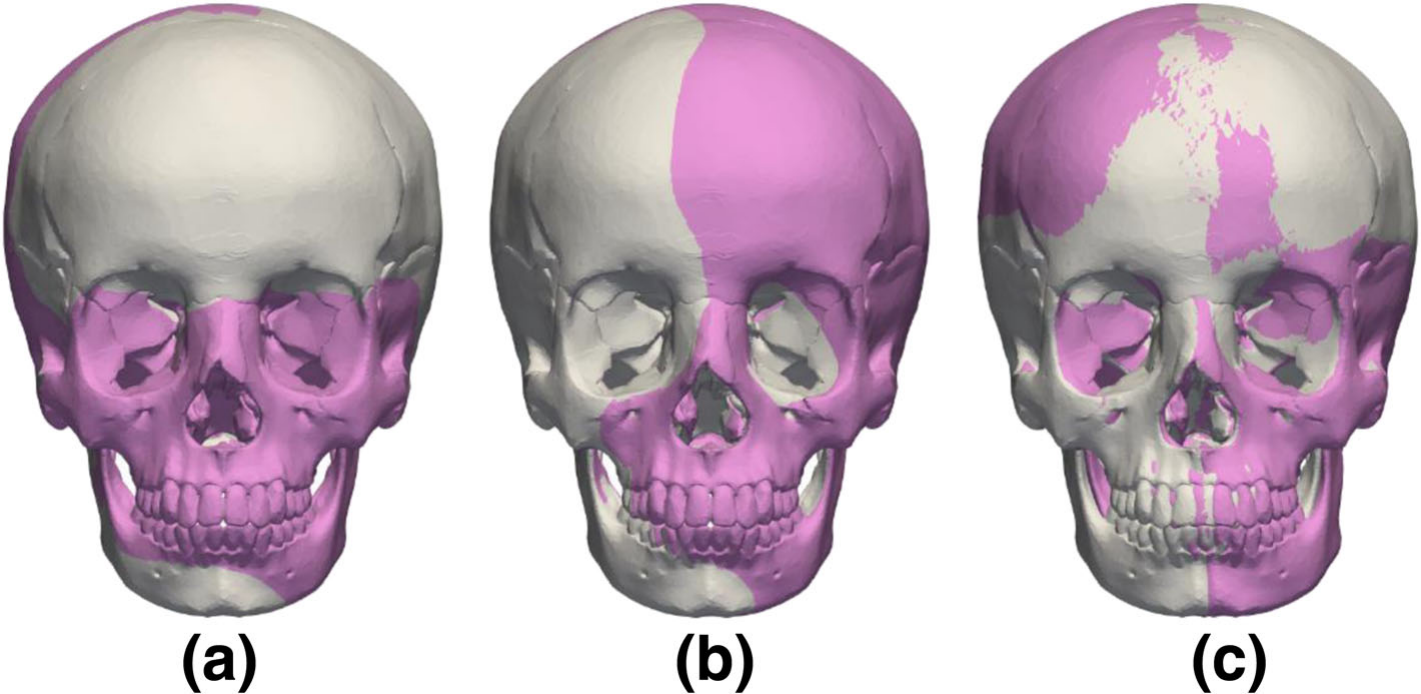
Final alignment between the gold standard and target model with: **a** protrusion of the midface, **b** unilateral protrusion of the face, and (**c**) lateral displacement of the mandible (gold standard model in grey and facial asymmetric model in purple)

The alignment of the asymmetric models showed good agreement with the theoretical models (Fig. 6a-c), and good agreement was obtained even when the two landmarks N and Pog did not lie in the symmetry plane (Fig. 3c and 6c). Table 4 summarizes the distances between cephalometric landmarks for each independent facial asymmetry model. The errors in the quantification of facial asymmetry range from − 11.34% for asymmetry 2, to < 10% for asymmetry 1 and < 5% for asymmetry 3 (Table 4).

**Table 4:** Distance between displaced landmarks in the gold standard model and the facial asymmetry warped models (all values in millimetres)

Model n#	Distance	Cg-Cg^a^	IN-IN^a^	A-A^a^	lOr-lOr^a^	rOr-rOr^a^
1	Initial	2.9375	0.8662	2.8403	0.9399	1.2577
	Final	2.9486	0.8784	2.7976	1.0274	1.3432
	Diff.	0.0112	0.0121	−0.0426	0.0875	0.0854
	%	0.38	1.39	−1.50	9.31	6.79
2	Distance	IN-IN^a^	A-A^a^	lOr-lOr^a^	rOr-rOr^a^	lEc-lEc^a^
	Initial	0.8348	2.8129	1.0060	3.0186	1.8111
	Final	0.7532	2.6933	1.0340	2.9539	1.6050
	Diff.	−0.0816	−0.1196	0.0279	−0.0647	−0.2061
	%	−9.77	−4.25	2.77	−2.14	−11.38
3	Distance	IN-IN^a^	A-A^a^	lGo-lGo^a^	rGo-rGo^a^	Me-Me^a^
	Initial	1.0050	2.9302	6.9993	6.9993	7.0037
	Final	1.0293	2.9564	7.2544	7.2550	7.0897
	Diff.	0.0242	0.0262	0.2551	0.2557	0.0860
	%	2.41	0.89	3.64	3.65	1.23

^a^Landmarks in the theoretical/original model; rOr stands for right orbitale landmark and lOr for left orbitale landmark

## Discussion

3D medical imaging techniques offer the opportunity for more accurate evaluation of the craniofacial anatomy, however most assessment protocols are still based on the concepts of 2D cephalometry. Here, a protocol for general 3D cephalometric analysis is proposed, which aims firstly to accurately estimate facial symmetry and the NHP and secondly to use the basicranium to superimpose other morphologically different skulls. A phantom study was conducted to quantify the level of accuracy and reliability with which small morphological differences within or between subjects can be evaluated. Since the measured differences between the *gold standard* and *warped models* is one to two orders of magnitude smaller than the initial perturbation, the facial growth and asymmetry assessments were assumed to be accurate and therefore the null hypothesis was rejected.

According to the results obtained, the protocol provides: (1) consistent definition of the craniofacial symmetry plane and the *true* vertical direction, and (2) a method for consistent alignment between different skulls, which is independent of the standard cephalometric landmarks. The true vertical direction is given by two user-defined landmarks (here the N and Pog), however these are internally corrected once the true symmetry plane is computed. To consistently align different skulls (for instance, of the same subject but at different time points), the basilar surface is used as the reference structure, instead of the geometrical centre of the sella turcica (Sella point (S)). In previously reported studies, the estimation of the symmetry plane was accomplished through the identification of multiple anatomical landmarks. Landmarks that were then mirrored about an arbitrary plane and matched using Procrustes superimposition [23–25]. Here, a global solution for the definition of the symmetry plane is proposed, simply by taking advantage of the 3D skull data and the properties of both PCA and ICP methods.

The PCA aligns the skull according to the main axes of variation and can be made relatively insensitive to any natural facial asymmetry by reflecting the skull along the initial estimate of the symmetry plane (section 2.1 step 2) and re-applying the PCA method to the matched original and mirrored skulls (section 2.1 step 5). It is important to stress that the definition of the symmetry plane is only as accurate as the ability to match the original and the mirrored models [18]. To obtain a better match between original and mirrored skulls the ICP method is applied. A similar approach was proposed in [26] to estimate the facial symmetry plane. Here, the procedure is landmark free by testing the eigenvector ***λ***_*i*_ that minimizes the root-mean-squared error (RMSE) of the Euclidean distances between the nearest points in the original and reflected models. The algorithm performed consistently for both full and partial skull data (Table 2).

In the definition of the NHP and with the newly defined symmetry plane, new estimates of the N and Pog landmarks and N-Pog line/plane are computed by estimating the nearest node to the landmark projection along the symmetry plane. However, it is important to stress that in the presence of a mandibular asymmetry (Fig. 3c) the identification of the nearest node along the symmetry plane would not achieve accurate results in the identification of the new Pog position. In this extreme scenario, the algorithm still produces an accurate estimate of the facial symmetry. This shows not only that the independence of the algorithm from user-defined landmarks, but also its usefulness for the management of patients with chin deviation [27]. In [26] an average angular deviation of 0.028° (0.014) was obtained between the estimated and the true symmetry plane. With the proposed procedure the maximum coronal deviation from the true symmetry is 0.30° (NHP recovery 15 trials, Table 2), and an upper limit of 0.36° of error in the coronal plane obtained for facial asymmetry 2 (Table 2).

In facial growth and asymmetry quantification, the final skull alignment is defined by the facial symmetry procedure and the ICP with weights, which minimizes distances across the basilar surface. Since in adolescence and adulthood there is a stabilization of the craniofacial base and foramen magnum [12], this anatomical region was considered suitable for 3D skull alignment. Similarly to the NHP recovery, the final alignment was independent of the user-defined positions of both the N and Pog landmarks. The advantage of using the basilar surface becomes evident in the presence of both facial growth (Fig. 5a-b) and facial asymmetry (Fig. 6a-c), in particular when the Pog landmark is not in the symmetry plane (Fig. 6c).

The alignment protocol allowed an accurate quantification of facial growth, with an error that ranged from − 11.38 to 9.31% for 2-years and from − 11.38 to 2.77% for 4-years facial growth (Table 3). Good skull alignment was also obtained when considering facial asymmetry. The linear measurements between landmarks showed that the error ranges from − 1.50 to 9.31% for asymmetry 1, from − 11.38 to 2.77% for asymmetry 2 and from 0.89 to 3.65% for asymmetry 3 (Table 4). The results show that the protocol might be useful to quantify slight asymmetrical variations in craniofacial anatomy, especially in patients with cleft-lip and palate. Facial asymmetry in patients with cleft-lip and palate typically range between 0.79 (0.23) mm in the lower face up to 1.15 (0.44) mm in the midface [28]. All the errors in asymmetry estimation are below the aforementioned values (Table 4).

The new protocol to determine the true craniofacial symmetry plane has numerous clinical applications, namely (1) as a part of a *true* 3D cephalometric analysis protocol to assess craniofacial morphology, growth and asymmetry as demonstrated above; (2) determination of the orientation of dental occlusion plane relative to the craniofacial morphology; (3) quantification of mandibular asymmetry and biomechanical investigations in masticatory asymmetry [29], (4) it may also be useful for virtual surgical planning of mandibular reconstructions [30], and mandibular implant development [31]. The protocol is landmark free and allows the reliable determination of the true craniofacial symmetry against a *gold standard model*. It can be combined with other mid-plane cephalometric landmarks (such as N and Pog) to position the skull in a standard anatomical position. The alignment error was quantified for both NHP, facial growth and facial asymmetry and was evaluated against the ideal alignment defined by a clinical expert. The results obtained demonstrate that symmetry alignment is accurate and independent of errors associated with the user-defined landmarks. In addition, the correct alignment of morphologically disparate skulls can be accomplished using the basilar surface instead of the traditional sella turcina (S) landmark. This is an important advance alongside the contemporary trend towards 3D imaging and the need to be able to reliably quantify longitudinal changes in 3D, with landmark free superimposition producing greater precision and reliability than previously used methods. With the craniofacial images being derivable from MRI, it is also non-invasive, clinically useful in orthodontics and maxillofacial surgery, versatile and applicable to other anatomical areas and even other fields of interest.

## Conclusions

The protocol proposed for 3D skull alignment is fully automated and produces an accurate and landmark free estimation of the true symmetry plane of the human skull. It also allows the accurate alignment of the skull in the NHP, taking into account the true symmetry plane of the skull and two used-defined landmarks along the facial profile that specify the true vertical direction. After the definition of the true symmetry plane, the user-defined landmarks are internally corrected and the final alignment is completely independent of any user-defined data. The algorithm produces accurate quantifications of both facial growth and asymmetry, which renders it useful for clinical applications.
